# Neutral metalloaminopeptidases APN and MetAP2 as newly discovered anticancer molecular targets of actinomycin D and its simple analogs

**DOI:** 10.18632/oncotarget.25532

**Published:** 2018-06-29

**Authors:** Ewelina Węglarz-Tomczak, Michał Talma, Mirosław Giurg, Hans V. Westerhoff, Robert Janowski, Artur Mucha

**Affiliations:** ^1^ Department of Bioorganic Chemistry, Faculty of Chemistry, Wrocław University of Science and Technology, Wrocław, Poland; ^2^ Synthetic Systems Biology and Nuclear Organization, Swammerdam Institute for Life Sciences, Faculty of Science, University of Amsterdam, Amsterdam, The Netherlands; ^3^ Department of Organic Chemistry, Faculty of Chemistry, Wrocław University of Science and Technology, Wrocław, Poland; ^4^ Institute of Structural Biology, Helmholtz Zentrum München-German Research Center for Environmental Health, Neuherberg, Germany

**Keywords:** metalloaminopeptidases, cancer, actinomycin D, phenoxazones, biological activity

## Abstract

The potent transcription inhibitor Actinomycin D is used with several cancers. Here, we report the discovery that this naturally occurring antibiotic inhibits two human neutral aminopeptidases, the cell-surface alanine aminopeptidase and intracellular methionine aminopeptidase type 2. These metallo-containing exopeptidases participate in tumor cell expansion and motility and are targets for anticancer therapies. We show that the peptide portions of Actinomycin D and Actinomycin X_2_ are not required for effective inhibition, but the loss of these regions changes the mechanism of interaction. Two structurally less complex Actinomycin D analogs containing the phenoxazone chromophores, Questiomycin A and Actinocin, appear to be competitive inhibitors of both aminopeptidases, with potencies similar to the non-competitive macrocyclic parent compound (*K*_i_ in the micromolar range). The mode of action for all four compounds and both enzymes was demonstrated by molecular modeling and docking in the corresponding active sites. This knowledge gives new perspectives to Actinomycin D's action on tumors and suggests new avenues and molecules for medical applications.

## HIGHLIGHTS

Human MetAP2 and APN are molecular targets of the anticancer drug Actinomycin D.

Actinomycins D and X_2_ are non-competitive inhibitors of both exopeptidases.

Simple heteroaromatic analogs share the activity with Actinomycin D.

Questiomycin A and Actinocin show competitive mechanism of inhibition.

## INTRODUCTION

**Figure 1:**
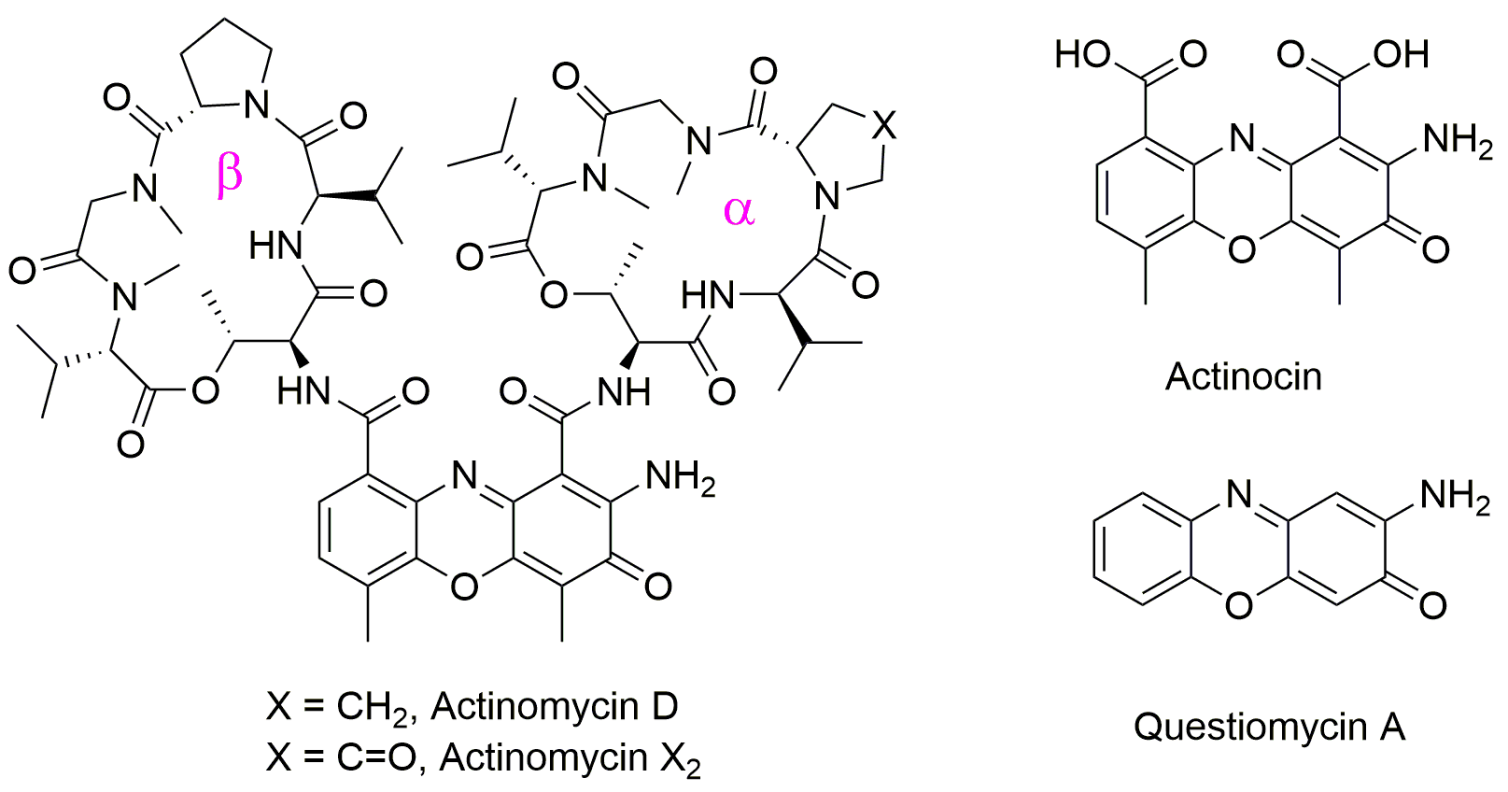
Structures of Actinomycin D, Actinomycin X2, Actinocin and Questiomycin A

Natural products are excellent leads for drug development. Particularly in the areas of antibiotic and anticancer therapies, natural products and their derivatives comprise a significant percentage of clinically used drugs [1]. Actinomycin D (ActD), also known as Dactinomycin, a small molecule naturally produced by *Streptomyces* bacteria [2], was the first antibiotic shown to have anticancer activity. Approved for medical use in the United States in 1964 and launched under the trade name Cosmegen [3], Dactinomycin has been mostly used as a remedy for a variety of mainly pediatric tumors, such as Wilms’ tumor, rhabdomyosarcoma and Ewing's sarcoma [4–9]. ActD is a neutral molecule consisting of a planar phenoxazone ring substituted with two carboxylate-linked cyclic pentapeptides (Figure 1). Its anticancer role has been associated with DNA functionality, leading to RNA, and consequently, protein synthesis inhibition [10]. ActD binds to double and single-stranded DNA (but not to RNA) with its phenoxazone ring intercalating with high specificity between GpC base pairs. Moreover, it stabilizes cleavable topoisomerase I and II complexes with DNA, in which the polypeptide lactone rings occupy a position in the minor groove of the DNA helix or the drug penetrates the topoisomerase-DNA binding area [11, 12]. After years of Actinomycin D usage, its action was also correlated with apoptosis; at low doses, the drug is a potent inducer of apoptosis [13, 14], triggering this process in normal and tumor cells, as well as in cells that rarely undergo apoptosis [15]. It was shown that the molecular mechanism of Actinomycin D-induced apoptosis was associated with activation of the apoptosis FAS surface protein, which is also known as CD95 (cluster of differentiation 95) [16], the non-mitochondrial stress-activated protein/Junmino-terminal kinases apoptotic pathway, and with increased expression of the apoptosis regulator BAX [13]. Cells inducing apoptosis in the immune response pathway via Actinomycin D may also be associated with the expression of the CD69 surface antigen [17]. Additionally, the presence of intracellular ATP is necessary for the induction of apoptosis by Actinomycin D [18, 19]. In recent years, intensive research in laboratories and clinics has focused on the correlation between Actinomycin D activity and cellular tumor antigen p53 functionality and expression [20, 21]. ActD induces p53 expression [20, 22]. Apoptosis in patients with mutated or deleted p53 suggests a p53-independent cell death mechanism [23]. However, the function of p53 was restored after low doses of Actinomycin D in various *TP53* wildtype tumor cell lines [22, 24].

2-Amino-3*H*-phenoxazin-3-ones missing the cyclic pentapeptide fragments, such as Actinocin and Questiomycin A (Figure 1), are also naturally produced by *Streptomyces* [25]. They occur in nature as pigments, fungal metabolites and allelo-chemicals [26–28]. Compounds composing the 2-aminophenoxazin-3-one skeleton exhibit antitumor, antimicrobial, and antiviral activities *in vitro* and *in vivo* [29–33]. Soon after the discovery of Actinomycin D and its properties, the anticancer actions of Questiomycin A were also described [31], but the success of Actinomycin D turned attention away from its simpler analogues.

Human alanine aminopeptidase (*Hs*APN) and methionine aminopeptidase type 2 (*Hs*MetAP2) are representative metalloproteases that have been targeted as a therapeutic intervention against pathological disorders. Among others, they participate in proteolytic pathways associated with angiogenesis, apoptosis and proliferation, which makes them interesting objects of oncological research [34–36]. MetAP2 is the best recognized member of the three known intracellular methionine aminopeptidases that catalyze the removal of the N-terminal methionine residue from a number of nascent proteins. This step is required before folding into their functional forms [35, 37]. The enzyme binds two cobalt [37] or manganese ions [38] in its active site. As higher MetAP2 expression was observed in tumor (mesothelioma, [39] neuroblastoma, [40] and colorectal carcinoma [41]) cells compared with normal cells, the enzyme serves as a target for anti-angiogenic compounds of natural origin, such as fumagillin and ovalicin [42]. Selective inhibition of MetAP2 stops vascularization and tumor growth in animal models [43, 44].

Aminopeptidase N is a membrane-bound, zinc-dependent metalloproteinase also known as CD13 (cluster of differentiation 13) [45]. APN specificity is directed towards the cleavage of a neutral or basic amino acid from the *N*-terminal position of peptides and proteins [46]. In contrast to MetAP2, its active site is accessible to the exterior environment of the cell and the cell membrane anchoring its N-terminus [47]. Aminopeptidase N is described as a moonlighting protease with multiple functions, including antigen presentation. It is also a receptor for some human viruses, e.g., coronaviruses. These functions are connected not only to peptide cleavage but also to endocytosis, signaling and ligation or the inhibition of its enzymatic activity, which might result in complex and systemic effects [48]. APN overexpression has been observed in tumor cells from melanoma [49], renal [50], colon [51], gastric [52, 53], pancreatic [54], and thyroid [55] cancers and on leukemic blasts in acute myeloid leukemia [56]. Compelling studies have implicated APN protease activity in tumor-associated processes, particularly angiogenesis, apoptosis and metastasis [57–59]. Here, we show that Actinomycin D and its analogues inhibit human MetAP2 and APN.

## RESULTS AND DISCUSSION

**Figure 2:**
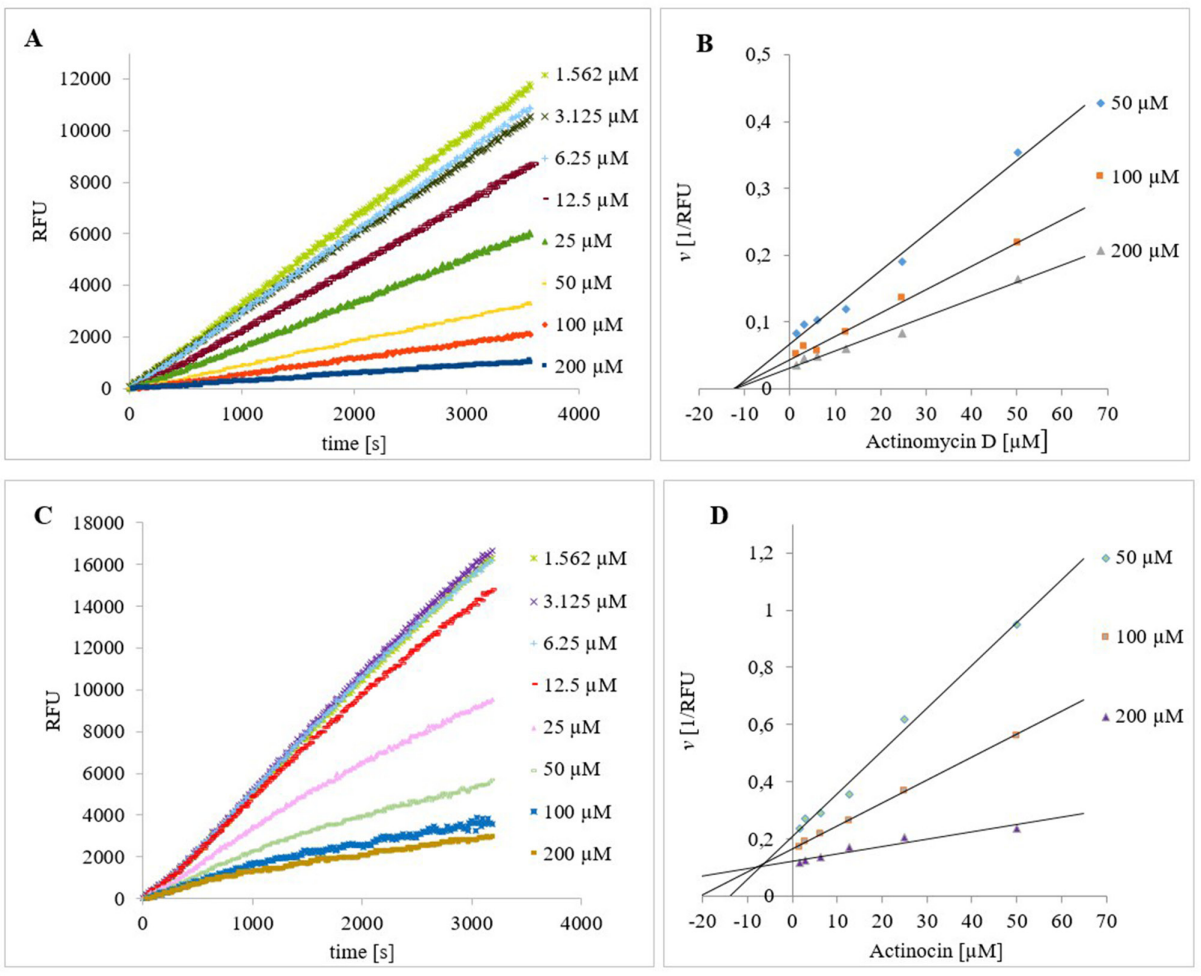
Progress curves for the hydrolysis of Ala-AMC by *Hs*APN in the presence of increasing concentrations of **(A)** Actinomycin D and **(C)** Actinocin. The final concentrations of Ala-AMC and *Hs*APN were 50 μM and 1 nM, respectively. Dixon plots of the inhibition of *Hs*APN by **(B)** Actinomycin D and **(D)** Actinocin in the presence of substrate and inhibitor at different concentrations.

Developments in synthetic ligands of metallo-aminopeptidases, substrates and inhibitors, indicated that not only amino acid and peptide mimetics but also heterocyclic compounds may effectively interact with these enzymes [46, 60–63]. Inspired by these accounts we have suggested the 2-amino-3*H*-phenoxazin-3-one scaffold as capable of binding to the aminopeptidase active sites. Indeed, the well-known antibiotic and anticancer drug Actinomycin D inhibits the two aminopeptidases *Hs*APN and *Hs*MetAP2 with inhibition constants approximately 10 μM (Table 1 and Figure 2A). Kinetic studies allowed us to determine the mechanism of inhibition of Actinomycin D as non-competitive, with steady-state binding being achieved immediately (Figure 2B). Comparable results (within the experimental error) were obtained for Actinomycin X_2_, a structure closely related to ActD. A minor variation in the amino acid composition of the α peptide side chain (Figure 1) did not influence their binding affinities. Despite structural similarity to ActD medicinal properties of Actinomycin X_2_ have been scarcely investigated. For example, Actinomycin X_2_ was reported to show higher cytotoxicity of the human leukemia cell line HL-60 (*LC*_50_ = 8 nM, *LC*_50_ is the lethal concentration for a half of population) than Actinomycin D (*LC*_50_ = 45 nM) [64]. Further studies proposed Actinomycin X_2_ as an inducer of apoptosis of human prostate cancer cells through the mTOR pathway compounded by miRNA144 [65].

**Table 1:** Inhibitory activities of Actinomycin D and its analogs Actinomycin X_2_, Actinocin and Questiomycin A toward two human neutral aminopeptidases (*Hs*APN and *Hs*MetAP)

Compound	*Hs*APN		*Hs*MetAP	
	*K*_i_ [μM]^*^	*IC_50_* [μM]	*K*_i_ [μM]^*^	*IC_50_* [μM]
Actinomycin D	11.9 ± 1.1		7.37 ± 0.56	
Actinomycin X_2_	12.2 ± 1.4		8.33 ± 0.75	
Actinocin	6.04 ± 0.51	7.25 ± 1.2	2.04 ± 0.23	2.34 ± 0.62
Questiomycin A	52.5 ± 2.7	63.0 ± 3.2	30.4 ± 3.2	34.9 ± 3.2

The inhibitors were screened in TRIS buffer. The release of the fluorophore was monitored continuously. The linear portion of the progress curve was used to calculate the velocity. Each experiment was repeated at least three times and the results are presented as the average with standard deviation. For more details, please see the Materials and methods section.

^*^Calculated based on the Cheng-Prusoff equations for non-competitive and competitive inhibitors [66].

The analogs that do not possess the macrocyclic polypeptide fragments, Actinocin (Figure 2C and 2D) and Questiomycin A, exhibited competitive inhibition mechanisms for each aminopeptidase. Regarding their potency, they differed significantly from one another. Actinocin was twice as effective as Actinomycin D/X_2_ in the case of *Hs*APN and four times as effective in the case of *Hs*MetAP2. In contrast, Questiomycin was 10 times less effective; the unsubstituted 3-aminophenoxazone ring gave rise to a five-fold decrease in activity for *Hs*APN and *Hs*MetAP2 compared to the lead compound. Cytotoxicity of Actinomycin D and Questiomycin A reported in the literature is visibly different to each other and to some extend corresponds with these results. 2-Aminophenoxazine-3-ones induced apoptosis in many types of cancer cells [67–75], however, in comparable cases of human acute B- and T-lymphoblastic leukemia, cervix carcinoma and larynx carcinoma, the cytotoxicity of Questiomycin A is up to three orders of magnitude lower (*IC*_50_ = 1-3 μM) than Actinomycin D (*IC*_50_ = 1-8 nM) [67].

**Figure 3:**
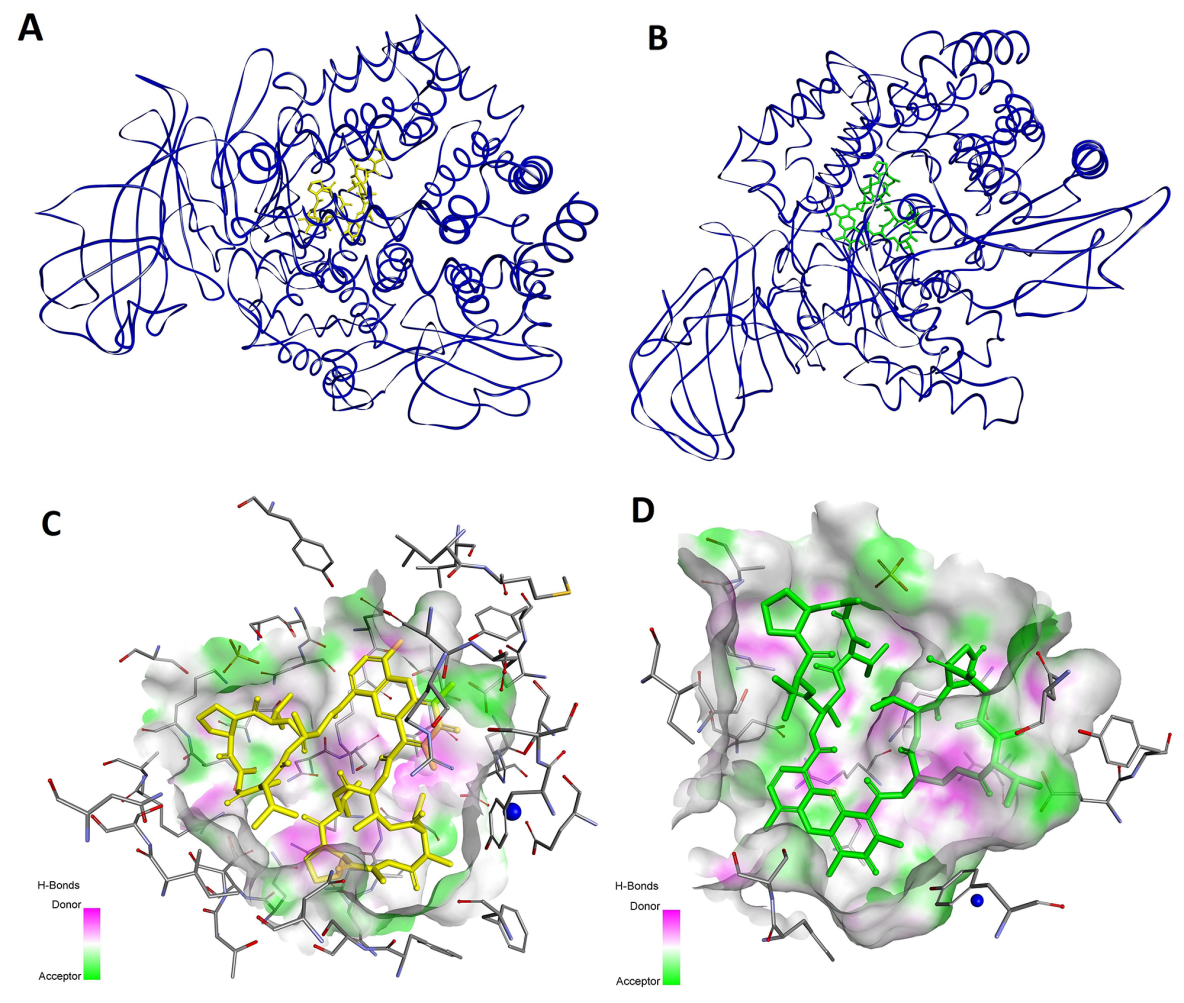
Modeled mode of binding for Actinomycin D and Actinomycin X_2_ to human alanine aminopeptidase (pdb code: 4FYT) [76] **(A** and **B)** The overall views. **(C** and **D)** Interactions with the amino acid residues in the inner cavity. Inhibitor and enzyme amino acid residues are shown as sticks, with yellow for the inhibitor. The catalytic zinc ion is shown as a dark blue sphere. The enzyme surface is colored according to the character of the hydrogen bonds formed.

**Figure 4:**
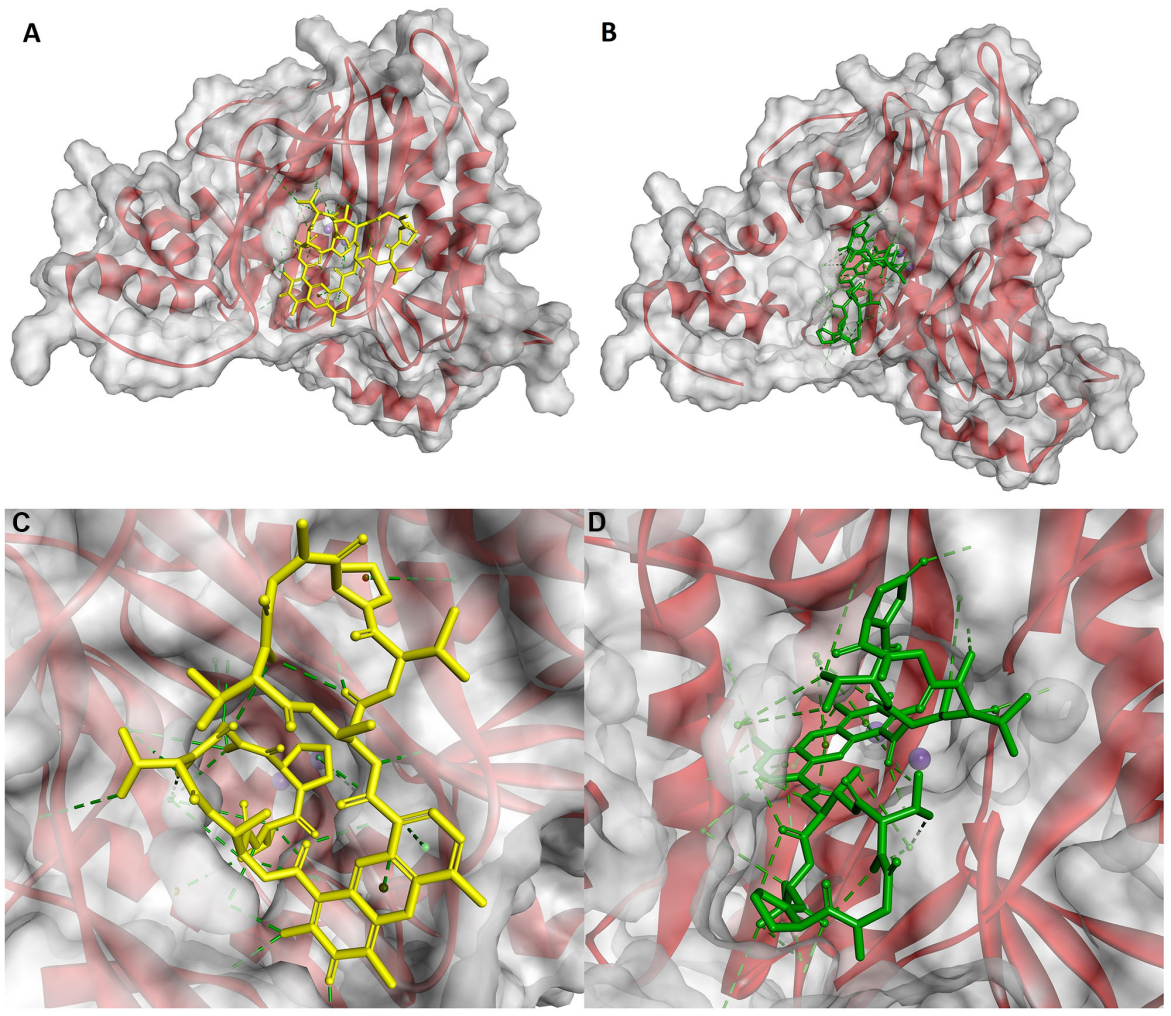
Modeled mode of binding for Actinomycin D and Actinomycin X_2_ to human methionine aminopeptidase (pdb code: 5D6E) [77] **(A** and **B)** The overall view. **(C** and **D)** Interactions with the amino acid residues at the entrance to the active site. Inhibitor and enzyme amino acid residues are shown as sticks, with yellow for the inhibitor. The cobalt ions are shown as dark purple spheres. Ligand interactions are shown as green lines.

The mechanisms of binding for each inhibitor must be significantly different at the molecular level due to the fundamental variations in their structures. This issue was studied in additional detail by molecular modeling. The modeled binding of ActD/Actinomycin X_2_ with *Hs*APN (Figure 3) and *Hs*MetAP2 (Figure 4) showed complex sets of contacts based on typical peptide-protein interactions at different conformations. In the case of APN [76], both drugs most typically penetrated the interior of the enzyme and filled the characteristic central cavity, which was apt to accept large peptide substrates (Figure 3A and 3B). The modeled assembly was generally stabilized by a hydrogen bonding network (Figure 3C and 3D), particularly well-defined for Actinomycin X_2_.

Arg381 of *Hs*APN has been identified as a residue highly involved in binding. For Actinomycin D its terminal guanidine nitrogen atom forms a convenient hydrogen bond bridge between both C=O groups of the heteroaromatic ring-substituting carboxyamides (2.91 Å and 3.34 Å, respectively, Figure 3C). In contrast, for the most favorable Actinomycin X_2_ conformation (Figure 3D), the corresponding N atom of Arg381 is engaged in interactions with the α cyclic peptide *via* two tight contacts with C=O of D-Val (2.54 Å) and Thr (2.61 Å). The extra oxygen atom of oxoPro is pointed towards the α-nitrogen of the same enzyme residue (3.29 Å). The β peptidyl ring of the inhibitor is also favorably bound by the guanidine residue of Arg829, with distances 2.76 Å and 2.81 Å to C=O of Thr and *N*-MeVal, respectively. The Thr residue is in additional contacts with the hydroxyl group of Ser861 (2.64 Å, *via* C=O) and the carboxylate of Asp855 (2.90 Å, *via* N-H). Finally, the amino group of phenoxazinone interacts with -OH of Tyr477, a group which is involved in the transition state stabilization during the natural substrate cleavage.

For MetAP2 [77], protein-Actinomycin D/X_2_ interactions mainly involved the surface of the enzyme (Figure 4A and 4B). The inhibitors are favorably located at the entrance of the active site, thereby blocking substrate inclusion and processing. In the lowest energy conformation, moieties of the cyclic peptide α of Actinomycin D are partially inserted into the active site cavity and surrounded by the hydrophobic enzyme residues: His231, Leu328, His331, Ile338, Pro445 and Leu447 (Figure 4C). The heterocyclic system is exposed to the outside and hardly contributes to this lipophilic binding. Nevertheless, the functional groups of 2-amino-3*H*-phenoxazin-3-one form a set of hydrogen bond contacts. The 2-amino group is bridged between the carboxylate of Asp442 (2.81 Å) and the hydroxyl O atom of Tyr444 (2.94 Å). The latter is also in a favorable position to N-H of d-Val of the cyclic peptide α (2.95 Å). In addition, the 3-oxo group of the inhibitor forms a contact with the N-H of Phe387 (3.10 Å). The β peptidyl ring of Actinomycin D interacts less tightly with the enzyme and only two specific hydrogen bonds are recognized. They involve the Thr residue, the C=O group binds with the side chain -NH_2_ of Asn329, and N-H with the hydroxyl O atom of Thr343.

For Actinomycin X_2_ the overall ligand-enzyme architecture is somewhat different as the β ring and the heterocyclic system are mostly involved in hydrophobic interactions with Phe219, His231, Ile338, His339, Phe387, His382, Met384, Ala414 and Leu447 of the protein surface (Figure 4D). This fit is emphasized by hydrogen bond contacts of the hydroxyl group of Tyr444 bridged between N-H and C=O of Thr (2.81 Å and 3.06 Å, respectively), and the guanidino group of Arg337 with C=O of D-Val (2.63 Å). The α peptidyl ring of the ligand forms two specific interactions which involve Asn327 and Asn329 (the side chain -NH_2_) of the protein, and oxoPro and *N*-MeVal (oxo/C=O, 2.75 Å and 2.89 Å, respectively) of Actinomycin X_2_.

**Figure 5:**
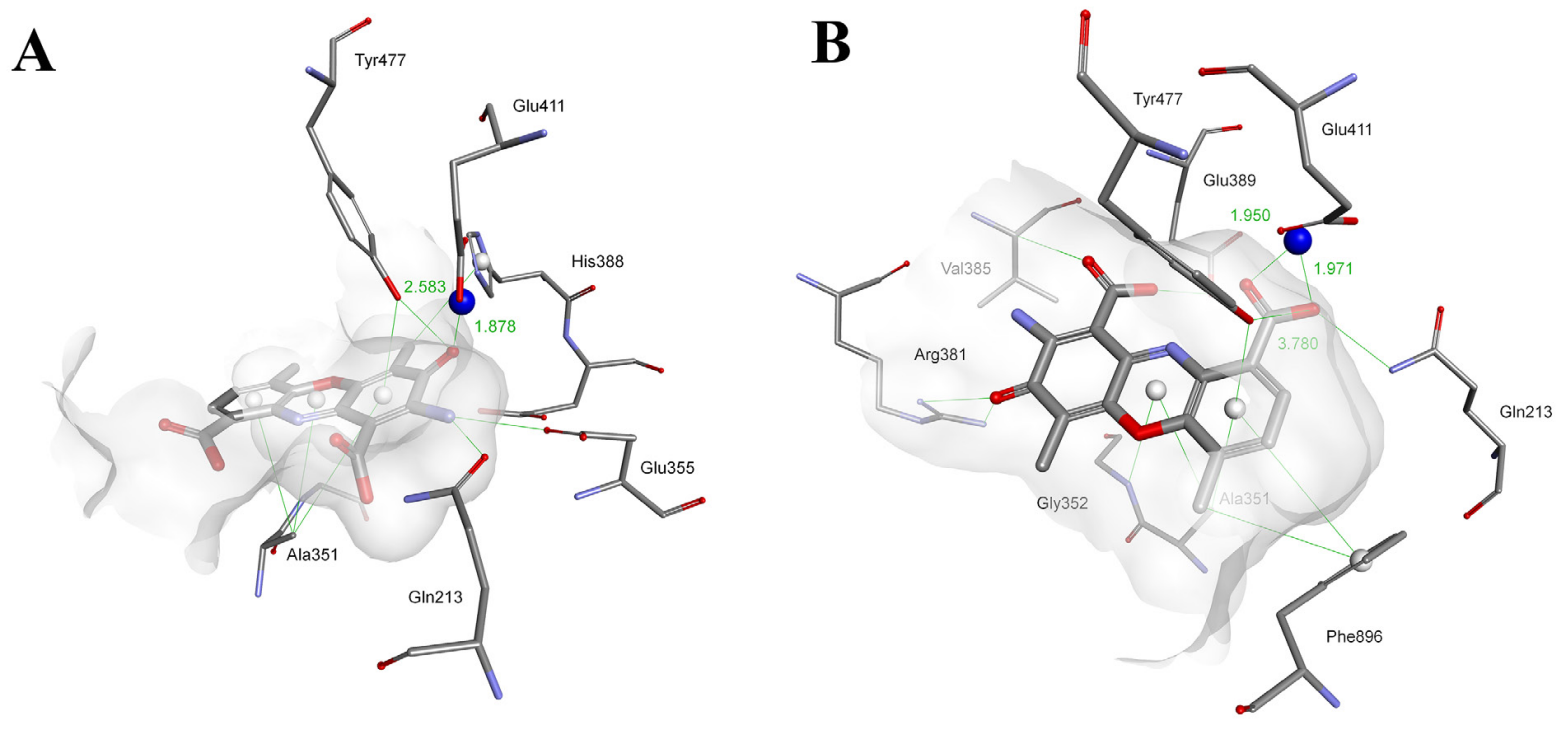
Modeled modes of binding for Actinocin to human alanine aminopeptidase **(A)** Interaction of the carbonyl oxygen with the zinc ion. **(B)** Interaction of the carboxyl group with the zinc ion. Inhibitor and enzyme amino acid residues are shown as sticks. The catalytic zinc ion is shown as a dark blue sphere. Hydrogen bonds, interactions with metal ions and hydrophobic contacts are shown as green lines.

**Figure 6:**
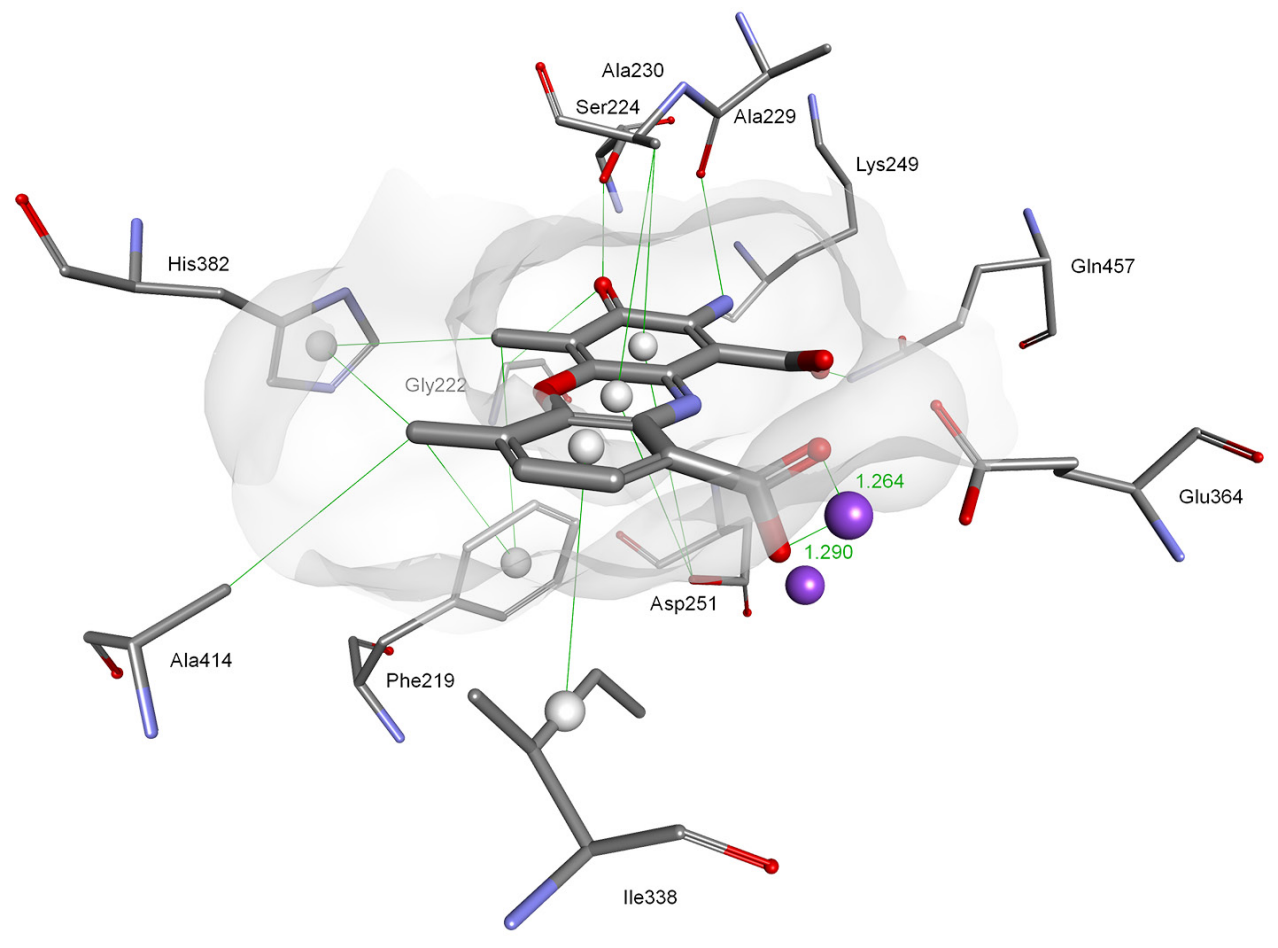
Modeled mode of binding for Actinocin to human methionine aminopeptidase 2 Inhibitor and enzyme amino acid residues are shown as sticks. Cobalt ions are shown as dark purple spheres. Hydrogen bonds, interactions with metal ions and hydrophobic contacts are shown as green lines.

Elimination of the cyclic peptide fragments from the structure of Actinomycin D/X_2_ allowed the resulting Actinocin to penetrate much further into the active sites of the studied metallopeptidases and to act as a classical competitive ligand by interacting with the metal ions and blocking the catalytic function (Figures 5 and 6). For human APN, the heteroaromatic ring is conveniently positioned in the hydrophobic environment while a functional group oxygen atom is directed towards the Zn ion. Interestingly, flipping the molecule does not seem to disturb the preferential binding. The highest rating docking score was shown for the conformation where the carbonyl oxygen atom is involved in an interaction with the metal cation (1.88 Å, Figure 5A). The oxygen was also in a tight hydrogen bond proximity to the Tyr477 -OH group (2.58 Å). These two contacts and the stabilization of the neighboring amino group by Glu355 (2.88 Å) and Gln213 (3.14 Å), similar to the peptide N-termini, closely resembles the substrate-enzyme complex, including the carbonyl positioning for the nucleophilic water attack. The zinc bidentate complexation with the 9-carboxylate group (1.95 Å and 1.97 Å) was an alternative thermodynamically favored conformation (Figure 5B). In this case, the 3-oxo group was in close hydrogen bond proximity to the Arg381 guanidine nitrogen atoms (2.65 Å and 2.68 Å, respectively). The 1-carboxylate also formed a potential O-H^…^O=C contact with the Glu389 side chain group (2.79 Å).

Similarly, the main driving force for the binding of Actinocin to *Hs*MetAP was a tight bidentate complexation between one of the cobalt ions and 9-carboxylate (1.26 Å and 1.29 Å, Figure 6). This interaction was further strengthened by the close three-dimensional fit of the molecule in the active site provided by hydrophobic contacts with Phe219, Ala230, Ile338 and His382 and hydrogen bonds. In particular, the latter involved interactions between the 2-amino group and the Ala229 (2.82 Å) and Lys 249 (2.30 Å) oxygen atoms, as well as the 3-oxo group and the Ser224 hydroxyl (2.41 Å). This set of favorable interactions gave rise to the highest theoretical docking score for Actinocin to MetAP and was consistent with the lowest obtained inhibition constant values.

**Figure 7:**
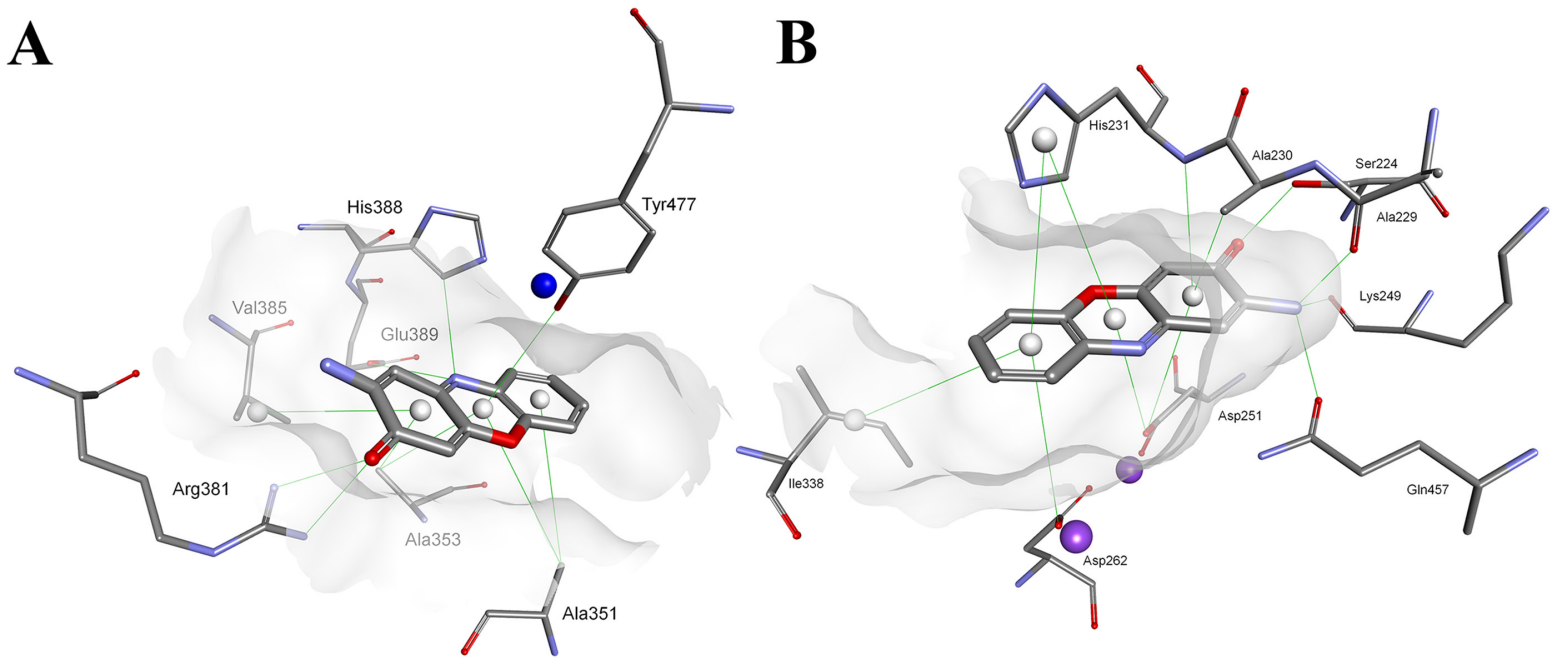
Modeled modes of binding for Questiomycin A to **(A)** human alanine aminopeptidase and **(B)** methionine aminopeptidase 2. Inhibitor and enzyme amino acid residues are shown as sticks. The catalytic zinc ion is shown as a dark blue sphere. Hydrogen bonds and hydrophobic contacts are shown as green lines.

Lacking both carboxylate groups, Questiomycin A loses the affinity of Actinocin to both APN and MetAP. The decrease in inhibitory activity is not dramatic, approximately one order of magnitude, but demonstrates that the metal-binding moieties are important but not critical for the structure and potency of a good ligand. Indeed, according to the molecular modeling results, the general fit of the heteroaromatic system and specific hydrogen bonds are reproduced for Questiomycin A in the active sites of the human metallopeptidases (Figure 7A and 7B). For *Hs*APN (Figure 7A) the 3-oxo group of Questiomycin A is firmly bridged between Arg381 guanidine nitrogen atoms (2.66 Å and 2.70 Å, respectively) while the heteroaromatic ligand core is nested in the hydrophobic environment of Ala351, Ala353, Val385 and His388. Similarly, the structurally simplest inhibitor reproduces interactions of the functional groups with MetAP. 2-Amino group is hydrogen-bonded with C=O of Ala229 (3.07 Å), Lys 249 (2.88 Å) and Gln457 (2.84 Å), while the 3-oxo group with the Ser224 hydroxyl (2.44 Å). The lipophilic contacts involve Ala230, His231 and Ile338. Taking in account these close similarities of Questiomycin A *versus* Actinocin binding, it can concluded that the difference in their activities are predominantly caused by the absence of metal coordination by carboxylates.

## PERSPECTIVE AND CONCLUSIONS

Actinomycin D is a long-known drug that was developed as an anticancer agent years before apoptosis and other cell death mechanisms and cancer progression were elucidated. It is still present in the World Health Organization's List of Essential Medicines [78], which contains the most effective and safest medicines required by any proper health system. While ActD is widely used in the treatment of cancer, intensive research on its mechanism of action, as well as on clinical trials with Actinomycin D in combination with other drugs, are progressing. At low concentrations, Actinomycin D inhibits proliferation and induces cell death in neuroblastoma cell lines, as well as tumor regression in xenograft tumor models. Cortes *et al*. proved experimentally that apoptosis is the major cell death mechanism caused by Actinomycin D treatment in p53wt cells, though less so in p53-deficient cell lines [79]. The combination of Actinomycin with drugs as Nutlin-3 [80] and the immunotoxin RG7787 [81] have improved the anticancer activities of the latter by activating apoptosis and p53.

Questiomycin A was demonstrated as costive agent of apoptotic cell death in the gastric and colon cancer cell line by dysregulating the function of mitochondria, and activating the caspase signaling [68, 69, 74]. Questinomycin A also induced cellular apoptosis by causing rapid intracellular acidification in several cancer cells [69, 70, 72, 74], generating reactive oxygen species in human lung adenocarcinoma cells and human glioblastoma cell line [71, 72] and by DNA laddering and upregulated Fas expression in mouse melanoma B16 cells [75]. Direct connection of Questiomycin with p53 still remains an issue to be elucidated. There are deep premises that it exists. The cell-surface receptor FAS, a member of the TNF-R family of receptors, is a key component of the extrinsic death pathway [82]. It has been reported that p53 can activate the extrinsic apoptotic pathway through the induction of genes encoding transmembrane protein FAS [83] that could be unregulated by Questiomycin A [75].

In contrast, aminopeptidases have also been subject to intensive research in oncological studies. Accordingly, in recent years, methionine aminopeptidase type 2, as well as alanyl aminopeptidase, have become extremely specialized targets for the development of new inhibitors [48, 58–63, 76, 77, 84, 85]. Most known MetAP2 inhibitors are irreversible binding synthetic analogues of fumagillin [77, 86], two of which, TNP-470 and Beloranib, have advanced to clinical trials. TNP-47 has been shown to inhibit angiogenesis *in vitro* and *in vivo*. It entered clinical development for cancer as an anti-angiogenic agent and has achieved Phase I/II trials for Kaposi's sarcoma, renal cell carcinoma, brain cancer, breast cancer, cervical cancer and prostate cancer. However, the trials have not led to its approval for clinical applications. Beloranib was originally designed as an angiogenesis inhibitor similar to TNP-470 for the treatment of cancer. Once the potential anti-obesity effects of MetAP2 inhibition became apparent during trials, clinical development began to focus on these effects, and Beloranib has shown positive results in preliminary clinical trials for this indication [87]. Moreover, there are tens of reversible inhibitors of both natural and synthetic origin. They include anthranilic acid sulfonamides [88], bengamides [89], benzoselnazalones [63] and compounds based on bestatin [90], 1,2,4-triazole [91], and pyrazolo[4,3-b]indole [92]. Research on new, reversible agents that are safe for organisms is ongoing.

The most known and widely studied reversible APN inhibitors, Bestatin and CHR-2797, also known under the commercial names Ubenimex and Tosedostat, respectively, have been investigated clinically for their anticancer effects. Ubenimex is approved for the treatment of lung cancer and nasopharyngeal cancer, and Tosedostat showed significant anti-leukemic activities in Phase I/II clinical trials. Currently, Phase I/II studies of Tosedostat in combination with other chemotherapeutic agents are in progress for AML and metastatic pancreatic adenocarcinoma [93]. Compounds with APN inhibitory activities improved the anti-metastasis and anti-angiogenesis effects *in vivo* [48, 94]. Additionally, intensive studies on enzyme structure specificity led to the development of highly active competitive inhibitors of APN, with inhibition constants in the nanomolar range. Phosphonic and phosphinic analogues of amioacid and dipeptides [95, 96], benzosuberone [61], naturally occurring curcumin [97] and even more recently, a cyclic peptide inhibitor [84] can be counted in this group of compounds.

The recent research on Actinomycin D highlights its huge therapeutic potential and suggests that low doses of this drug could be used in combination with other agents to take advantage of its unique activity and avoid non-specific effects. Nevertheless, there is no correlation between these properties and the inhibition of the neutral aminopeptidases MetAP2 and APN that is highly important in cancer progression. Blocking the activity of MetAP2 and APN with Actinomycin D or its analogs seems to be promising for the development of new generations of potent anticancer agents that would be implicated in different mechanisms of action and directed against multiple molecular targets. In view of the network nature of cancer [98], the use of combinations of inhibitors appears to be indicated, as well as alternative drugs with multiple specificities. Actinomycin D may prove to be such a drug for targeting a network rather than a single molecule. The discovery reported here may contribute to the development of better therapies.

## MATERIALS AND METHODS

### Materials and general methods

Actinomycin D was purchased as a lyophilized powder from Sigma-Aldrich (Poznan, Poland), Actinomycin X_2_ was purchased form Adipogen (Liestal, Switzerland). Actinocine and Questiomycin A were synthesized according to a previously described procedure [99]. Recombinant human APN and MetAP2 were purchased as a lyophilized powder from R&D Systems (Minneapolis, USA). Fluorogenic substrates Ala-AMC (L-alanine-4-methylcoumaryl-7-amide) and Met-AMC (L-methionine-4-methylcoumaryl-7-amide) were purchased from PeptaNova (Sandhausen, Germany). Spectrofluorometry was performed on a Spectra MAX Gemini EM fluorimeter (Molecular Devices, Sunnyvale, USA) in a 96-well plate format (Excitation = 355 nm, Emission = 460 nm). Calculations of the kinetic parameters were performed using the SoftMax Pro, GraphPad Prism and Microsoft Excel programs.

### Enzyme activation

Recombinant human APN enzyme was dissolved in 50 mM Tris-HCl buffer (pH = 7.0) and used directly in the kinetic experiments. Recombinant human MetAP2 was obtained as a lyophilized powder from R&D Systems. The enzyme was dissolved in 100 mM Tris-maleate buffer containing 0.1 mM CoCl_2_, 100 mM NaCl and 0.075 % v/v albumin at pH = 7.5 and incubated for 30 minutes. After this activation, the enzyme was used directly in the kinetic experiments.

### Inhibition constant determination

The inhibitors were screened against human APN and human MetAP2 at 37°C in the assay buffer as described above. For the steady-state measurements, the enzymes were preincubated for 30-60 min at 37°C with an inhibitor before adding the substrate (Ala-AMC, 40 μM for human APN or Met-AMC and 50 μM for MetAP2) to the wells. For the initial state measurements, the enzymes were preincubated for 15-30 min at 37°C before adding the substrate and inhibitor to the wells. The following conditions were used: (1) total volume 100 μL, (2) eight different inhibitor concentrations, and (3) enzyme concentration of 0.3 nM for human APN and 1 nM for MetAP2. The release of the AMC fluorophore was monitored. The linear portion of the progress curve was used to calculate the velocity. hPhePCH_2_Phe [62, 95] for APN and ebselen [63] for MetAP2 were used as a positive control. The inhibition mechanism was determined from the Lineweaver-Burk and Dixon plot (Figure 2B and 2D). The *IC*_50_ values were calculated from the reaction velocity to inhibitor concentration plot fitted to the equation log[I] vs. response. The *K*_i_ values were equal to the *IC*_50_ for non-competitive inhibitors [66] and were calculated for competitive inhibitors using the following equation: *K*_i_ = (*IC*_50_-[E]/2)/ (1 + (*[S]*/*K*m)) [66], where the *IC*_50_ is the concentration of the inhibitor that effected 50% inhibition, [S] and *K*_m_ are the substrate concentration and Michaelis constant in the absence of inhibitor, respectively, and [E] is the concentration of the enzyme.

### Molecular modeling

Molecular modeling studies were performed using the Biovia Discovery Studio program package (San Diego, Ca, USA). The crystal structures of human aminopeptidase N (CD13) in complex with amastatin (pdb id. 4FYT) [76] and methionine aminopeptidase type 2 (pdb id. 5D6E) [77] were used as the starting points for the calculations of the enzymes complexes with Actinomycin D, Actinomycin X_2_, Actinocin and Questiomycin A. The enzyme models were protonated at pH 7.5 and minimized with the CHARMM force field. The water molecules and former ligands were removed. New ligands were protonated at the same pH and the partial charges of all the atoms were computed using the Momany-Rone algorithm. Minimizations used the CHARMM force field with the Smart Minimizer algorithm up to an RMS gradient of 0.1 Å. Actinomycin D, Actinomycin X_2_, Actinocin and Questiomycin A were docked into the enzyme active center using the LibDock docking algorithm. The number of Hotspots was changed from 100 to 1000 (standard method). The ‘BEST’ Conformation Method was selected and the minimization of the results was achieved using the CHARMM force field.
